# A poor man’s BLASTX—high-throughput metagenomic protein database search using PAUDA

**DOI:** 10.1093/bioinformatics/btt254

**Published:** 2013-05-07

**Authors:** Daniel H. Huson, Chao Xie

**Affiliations:** ^1^Singapore Centre on Environmental Life Sciences Engineering, School of Biological Sciences, Nanyang Technological University, Singapore 637551, ^2^Center for Bioinformatics, University of Tübingen, 72076 Tübingen, Germany and ^3^Life Sciences Institute, National University of Singapore, Singapore 117456

## Abstract

**Summary:** In the context of metagenomics, we introduce a new approach to protein database search called PAUDA, which runs ∼10 000 times faster than BLASTX, while achieving about one-third of the assignment rate of reads to KEGG orthology groups, and producing gene and taxon abundance profiles that are highly correlated to those obtained with BLASTX. PAUDA requires <80 CPU hours to analyze a dataset of 246 million Illumina DNA reads from permafrost soil for which a previous BLASTX analysis (on a subset of 176 million reads) reportedly required 800 000 CPU hours, leading to the same clustering of samples by functional profiles.

**Availability:** PAUDA is freely available from: http://ab.inf.uni-tuebingen.de/software/pauda. Also supplementary method details are available from this website.

**Contact:** daniel.huson@uni-tuebingen.de or xiechao@bic.nus.edu.sg

In metagenomics studies, millions of DNA or cDNA reads are sequenced from environmental samples, and these are then analyzed in an attempt to determine the functional or taxonomic content of the samples (Handelsman *et al.*, 1998). An important computational step is to determine the genes or coding sequences present, which is usually done by aligning the sequences against a reference database of protein sequences. In most projects, BLASTX (Altschul *et al.*, 1990) has been the method of choice, despite the fact that running BLASTX requires thousands of CPU hours per million reads.

In the related area of read mapping, numerous methods have been developed to solve the problem of aligning sequencing reads against DNA reference sequences in a high-throughput manner (for example, Langmead and Salzberg, 2012). Using read mapping tools directly for analyzing complex metagenomes is problematic because environmental reads usually do not match existing genome reference sequences. Moreover, the underlying algorithms cannot easily be extended to protein sequences.

In this article, we present a new paradigm for the alignment of environmental sequencing reads called PAUDA, an acronym for ‘protein alignment using a DNA aligner’. It allows one to harness the high efficiency of DNA read aligners to compute BLASTX-like alignments.

The key idea is to convert all protein sequences into ‘pseudo DNA’, or ‘pDNA’ for short, by mapping the amino acid alphabet onto a four-lettered alphabet that reflects which amino acids are likely to replace each other in significant BLASTX alignments. A high-throughput sequencing read aligner, such as Bowtie2, is then used to compare pDNA reads with a pDNA database. For any match found, the participating pDNA sequences are translated back into protein sequences, and the corresponding protein alignment is calculated so as to determine statistical significance. The final output is a file of statically significant protein alignments in BLASTX format.

We have implemented this approach in a new software package called PAUDA. The package provides two scripts, pauda-build and pauda-run. The first script is run on the protein reference database and builds an appropriate index. The second script is run on a file of DNA reads and produces a BLASTX file as output. The two scripts use the Bowtie2 suite and a number of new Java programs that we have written. Bowtie2 can easily be replaced by some other method, if desired. An overview of the package is given in Figure 1.

**Fig. 1.**
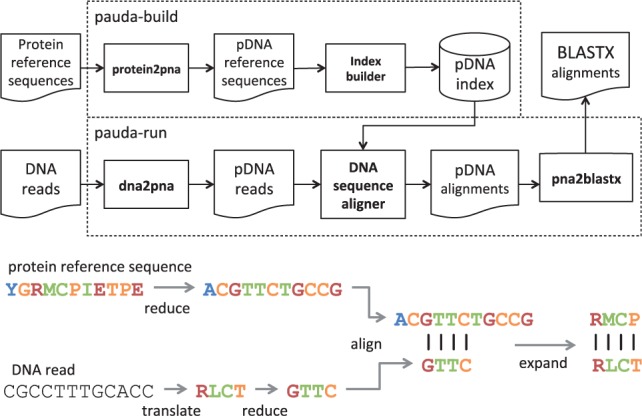
An overview of the PAUDA approach

Using Bowtie2 as the comparison engine, PAUDA runs ∼10 000 times faster than BLASTX, while assigning about one-third as many reads to KO groups. Because of the huge computational burden of running BLASTX on a large dataset, BLASTX is rarely run to completion; therefore, the key question is how many reads can be assigned per hour. PAUDA assigns ∼3000 as many reads as BLASTX does, per hour.

Mackelprang *et al.* (2011) present a taxonomic and functional analysis of 12 permafrost datasets. Reanalysis of their data, a comparison of 246 million Illumina reads with the KEGG database (Kanehisa and Goto, 2000), takes ∼2 h on a single workstation (64 cores, 512 GB of main memory) using PAUDA, reproducing the main result of the article.

In addition, we applied an early version of PAUDA to an unpublished dataset consisting of all 2.9 billion reads of a whole HiSeq2000 run on a waste-waster sample, requiring ∼2 days on 150 cores, whereas RAPSearch2 (Zhao *et al.*, 2012) required 115 days.

We produced a benchmark dataset for comparing the performance of PAUDA, BLASTX and RAPSearch2 by taking the first 600 000 good quality reads from each of the 12 samples published in Mackelprang *et al.* (2011). We then ran all three programs on each of the 12 samples benchmark samples, comparing with the KEGG database. Running all samples in parallel on a single workstation using 48 cores, the runtime ranged from 7 min (PAUDA) to 7 days (BLASTX), Table 1. We used the metagenome analysis program MEGAN (Huson *et al.*, 2011) to assign reads to KEGG orthology (KO) groups based on their alignments.

**Table 1.** Alignment of 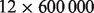 Illumina reads from permafrost data against the KEGG database

Method^a^	Time^b^	Speed-up^c^	Reads assigned^d^		KOs^e^		True KOs^f^	
PAUDA	7	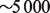	155 824	33%	4182	78%	1717	99.0%
RAPSearch2	510	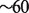	449 144	96%	5237	98%	1712	98.7%
BLASTX	30 240	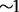	465 588	100%	5363	100%	1735	100%

^a^The method used.

^b^The number of wall-clock minutes required on 48 cores to process all 12 datasets.

^c^The speed-up over BLASTX.

^d^The number and percentage of reads that obtain a KO assignment.

^e^The number and percentage of different KO groups identified.

^f^The number of ‘true’ KO groups identified, defined as those that account for 99% of all reads with BLASTX hits. Percentages are in comparison with the results obtained by BLASTX. Note that half of the runtime reported here for PAUDA is start up overhead and on larger datasets the speed-up is 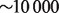.

Using PAUDA, the rate of assignment is 33% of that of BLASTX. In more detail, for alignments with a protein identity of 60, 70, 80, 90 and 100% the sensitivity is 35.1, 48.4, 61.6 and 78.5%, respectively. For alignments with identity <50%, the sensitivity is <8.1%.

For those reads for which both BLASTX and PAUDA are able to assign a KO group, the assignment differs in ∼2% of all cases. Assuming a false-positive error rate of 1% for the assignment of reads to KO groups, BLASTX identifies 1735 ‘true’ KO groups for this dataset that account for 99% of all reads with BLASTX hits. PAUDA identifies 99% of these. The number of reads assigned to individual KO groups by PAUDA and BLASTX is highly correlated, as shown in Figure 2. The Pearson correlation is 0.977 for linear read counts and 0.949 for log-transformed counts.

**Fig. 2.**
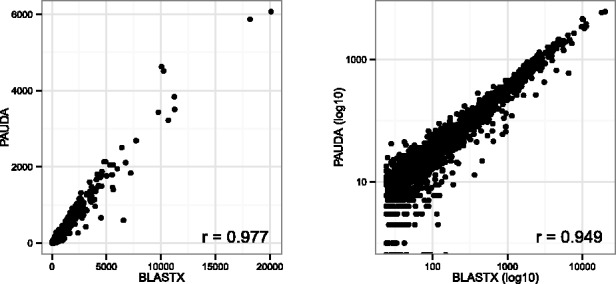
KEGG comparison of PAUDA and BLASTX. Left: Each true KO group is represented by a dot with coordinates that correspond to the number of reads assigned to the KO group by BLASTX (on the *x*-axis) and PAUDA (on the *y*-axis). Right: To show the low abundance KO groups more clearly, here, we plot the same data on a logarithmic scale

Using the LCA assignment algorithm as implemented in MEGAN, we also performed a taxonomic analysis of these datasets at a number of different taxonomic ranks. The results based on PAUDA and BLASTX are highly correlated, with a Pearson’s correlation coefficient *r* that ranges from 0.993 for the taxonomic rank of class to 0.953 for species. The corresponding range for log-transformed counts is 0.982–0.914.

To further illustrate the accuracy of PAUDA, we applied the program to all 12 permafrost samples in their entirety, in total comparing 246 million reads with the KEGG database.

A key result of (Mackelprang *et al.*, 2011) is that, on the one hand, two different frozen samples taken from the active layer of the permafrost have similar functional profiles, and that these change only little after thawing for 2 or 7 days. Although, on the other hand, two frozen samples obtained from the permafrost layer initially exhibit distinctive profiles that gradually become more similar during thawing. A PCoA analysis of Bray–Curtis distances (Mitra *et al.*, 2010) based on a PAUDA comparison of the data with the KEGG database delivers the same result in a small fraction of the computational time.

*Funding*: National Research Foundation and Ministry of Education Singapore under its Research Centre of Excellence Programme.

*Conflict of Interest*: none declared.
